# Expert consensus on osteoporosis risk management in patients with sleep disorders

**DOI:** 10.1038/s41413-026-00562-0

**Published:** 2026-08-13

**Authors:** Siqi Leng, Jinming Yang, Yuming Jin, Jie Shi, Ye Zhang, Junying Zhou, Lu Tan, Taomei Li, Peiyan Ni, Kejia Hu, Yuan Shi, Jiamin Liao, Yun Li, Pei Xue, Christian Benedict, Alexandros N. Vgontzas, Michael Furian, Talant Sooronbaev, Naima Covassin, Virend K. Somers, Zhoulong Yu, Hongqiang Sun, Xiao Tan, Jihui Zhang, Yun Kwok Wing, Yue Leng, Larry D. Sanford, Michael V. Vitiello, Katie L. Stone, Rong Ren, Lin Lu, Xiangdong Tang

**Affiliations:** 1 Sleep Medicine Center, Mental Health Center, National Center for Mental Disorders, Sleep Research Laboratory, West China Hospital, Sichuan University, Chengdu, China; 2 West China Biomedical Big Data Center, West China Hospital, Sichuan University, Chengdu, China; 3 Med-X Center for Informatics, Sichuan University, Chengdu, China; 4 National Institute on Drug Dependence and Beijing Key Laboratory of Drug Dependence Research, Peking University, Beijing, China; 5 Institute of Environmental Medicine, Karolinska Institutet, Stockholm, Sweden; 6 Sleep Medicine Center, Shantou University Medical College, Shantou, Guangdong China; 7 Department of Pharmaceutical Biosciences, Uppsala University, Uppsala, Sweden; 8 Sleep Research and Treatment Center, Department of Psychiatry and Behavioral Health, Pennsylvania State University, College of Medicine, Hershey, PA USA; 9 Department of Respiratory Medicine, University Hospital of Zurich, Zurich, Switzerland; 10 Swiss-Kyrgyz High Altitude Medicine and Research Initiative, Zurich, Switzerland; 11 Department of Respiratory Medicine, National Center for Cardiology and Internal Medicine, Bishkek, Kyrgyz Republic; 12 Swiss-Kyrgyz High Altitude Medicine and Research Initiative, Bishkek, Kyrgyz Republic; 13 Department of Cardiovascular Medicine, Mayo Clinic, Rochester, MN USA; 14 Peking University Sixth Hospital, Peking University Institute of Mental Health, NHC Key Laboratory of Mental Health (Peking University), National Clinical Research Center for Mental Disorders (Peking University Sixth Hospital), Beijing, China; 15 School of Public Health and Sir Run Run Shaw Hospital, Zhejiang University School of Medicine, Hangzhou, China; 16 Department of Clinical Neuroscience, Karolinska Institutet, Stockholm, Sweden; 17 Department of Psychiatry, Faculty of Medicine, The Chinese University of Hong Kong, Shatin, Hong Kong, Special Administrative Region of China; 18 Department of Psychiatry and Behavioral Sciences, University of California, San Francisco, CA USA; 19 Sleep Research Laboratory, Center for Integrative Neuroscience and Inflammatory Diseases and Biomedical and Translational Sciences, Macon & Joan Brock Virginia Health Sciences Eastern Virginia Medical School at Old Dominion University, Norfolk, VA USA; 20 Department of Psychiatry and Behavioral Sciences, University of Washington School of Medicine, Seattle, WA USA; 21 Research Institute, California Pacific Medical Center, San Francisco, CA USA; 22 Department of Epidemiology and Biostatistics, University of California, San Francisco, CA USA

**Keywords:** Osteoporosis, Diseases

## Abstract

A rate-limiting step in the prevention and early intervention of osteoporosis is identifying its asymptomatic onset. Accumulating evidence shows that sleep disorders are associated with an increased risk of osteoporosis. Given their early detectable and modifiable nature, integrating sleep disorder management into osteoporosis prevention and care pathways offers a novel approach for enhancing skeletal health. This expert consensus represents a collaborative effort by specialists in sleep medicine and orthopedics from across the world, integrating epidemiological, mechanistic, and interventional evidence to provide general guidance for prevention and clinical practice, and to foster multidisciplinary collaboration in the management of sleep disorders and osteoporosis.

## Introduction

Osteoporosis is a metabolic skeletal disease marked by diminished bone density and deterioration of bone microarchitecture, culminating in greater bone fragility and fracture susceptibility.^1^ As an age-related disorder, it has emerged as a global epidemic in the context of global aging, affecting approximately one-fifth of the general population worldwide, with substantial variation across countries and regions.^2,3^ Notably, osteoporosis exhibits a pronounced sex disparity, in which prevalence among females is approximately two to three times higher than in males, and this disparity is further amplified in the postmenopausal period.^3^ Fractures and their complications represent the major clinical outcome of osteoporosis, and lifetime risk estimates suggest that nearly 50% of women and 20% of men aged 50 years or older will experience at least one osteoporotic fracture.^4^ The global burden of osteoporosis continues to increase in parallel with population aging. According to the Global Burden of Disease Study, low bone mineral density (BMD) accounted for an estimated 438 000 deaths and 16.6 million disability-adjusted life years in 2019.^5^ Critically, about one-third of fall-related deaths were attributable to low BMD, underscoring the profound public health impact of osteoporosis and the urgent need for effective preventive strategies.^6^

Sleep has a critical role in promoting health.^7^ A sleep disorder is a clinically significant disturbance in sleep quantity, quality, timing, or the sleep-wake cycle, arising from dysregulated sleep-wake mechanisms or other physiological, developmental, or behavioral processes, that is of sufficient severity to result in significant distress, disability, or an elevated risk thereof, as manifested in conditions such as insomnia, circadian rhythm disruptions, and sleep-related breathing disorders such as obstructive sleep apnea (OSA).^8^ Insomnia is characterized by persistent difficulties with sleep initiation, maintenance, or early morning awakenings, with an estimated prevalence of 10%–20%, about half of which progress to a chronic course.^9^ Circadian rhythm sleep-wake disorders (CRSWDs), on the other hand, refer to conditions defined by misalignment between an endogenous circadian rhythm and external environmental or social demands,^10^ and their prevalence has increased in parallel with the rise of shift work, night shifts, and jet lag.^11^ In addition, OSA, marked by recurrent upper airway collapse during sleep leading to apneas or hypopneas, affects 6%–17% of the general adult population, with a higher prevalence among males.^12^ Collectively, these sleep disorders are highly prevalent and have been linked to dysfunction across multiple physiological systems, including cardiovascular, endocrine, metabolic, and neurological domains.^13–15^

The skeletal system and sleep health have long been regarded as distinct medical domains. However, accumulating epidemiological evidence indicates that major sleep disorders, including insomnia, CRSWDs, and OSA, are associated with increased risk of osteoporosis.^16–18^ Beyond nocturnal symptoms, sleep disorders involve multiple pathophysiological processes, such as neuroendocrine dysregulation, metabolic imbalance, chronic inflammation, and oxidative stress,^7,19^ which may directly or indirectly impair bone homeostasis, thereby accelerating bone loss and elevating osteoporosis risk. Given that sleep disorders are relatively amenable to early identification, screening, and intervention, whereas osteoporosis is typically asymptomatic in its early stages and progresses insidiously until clinical consequences such as fractures occur, reconceptualizing sleep disorders as potential “early warning” signs of osteoporosis carries substantial clinical and public health implications.^1^ Accordingly, integrating bone health screening and monitoring into the care of patients with sleep disorders, together with timely and effective management of sleep problems, may represent an innovative strategy to prevent or delay the onset and progression of osteoporosis.

This consensus therefore aims to comprehensively review the epidemiological, mechanistic, and interventional evidence linking sleep disorders to impaired bone homeostasis, and to provide practical guidance for integrating sleep disorder management into osteoporosis prevention and clinical care.

## Insomnia and osteoporosis

### Epidemiology

A growing body of epidemiological evidence demonstrates that insomnia may have a detrimental impact on osteoporosis. Sivertsen et al. conducted a longitudinal analysis of 24 715 participants from the large, population-based Nord-Trøndelag Health Study in Norway, revealing insomnia as an independent risk factor for self-reported osteoporosis.^20^ Similarly, a large-scale retrospective cohort study using data involving 134 070 participants, found that patients with insomnia had a significantly higher incidence of clinically diagnosed osteoporosis compared to those without insomnia.^21^ In a different population context, Cherian et al. carried out a prospective study among Indian postmenopausal females and reported insomnia as a predictive factor for clinically diagnosed osteoporosis.^22^ Likewise, Pengpid et al. found that insomnia was positively associated with self-reported osteoporosis among older adults (*n* = 2 863) in the Health, Aging and Retirement in Thailand study.^23^ Moreover, Rassow et al., in a population-based cohort study enrolling 1 037 participants in northeast Germany, observed positive associations between insomnia and lower sleep quality with self-reported osteoporosis.^24^ Furthermore, Zhao et al. utilized cohort data from the UK Biobank, comprised of 293 164 retired participants, and demonstrated longitudinal associations between insomnia and incident osteoporosis, as well as osteoporosis with pathological fractures.^25^

Inconsistent relationships between insomnia and BMD have been reported. Zhou et al.’s prospective cohort study, based on the UK Biobank, indicated that insomnia was negatively associated with total BMD, as well as falls and fractures.^26^ Similarly, the analysis by Cherian et al. demonstrated that, compared to participants exhibiting normal sleep, those with insomnia experienced a more significant decline in BMD at the femoral neck and lumbar spine.^22^ However, two cross-sectional studies performed by Niu et al. and Yamaura et al. found no significant associations between insomnia and BMD.^27,28^ Additionally, in the 10.7-year prospective Tasmanian Older Adult Cohort study, Pan et al. reported that self-reported interrupted sleep showed no association with BMD at the hip, spine, or total body, yet was independently associated with an increased risk of falls and fractures.^29^ Moreover, Cherian et al.’s reported that insomnia was associated with alterations in trabecular bone score (TBS), a validated indirect index of trabecular microarchitecture. Their findings further established a significant association between insomnia and bone turnover markers in Indian postmenopausal females, characterized by an increase in the bone resorption marker C-telopeptide of Type I Collagen (CTX) and a decrease in the bone formation marker procollagen type I N-terminal propeptide (P1NP).^22^ Comparably, an investigation conducted by Rassow et al. reported that higher scores on the Insomnia Severity Index were significantly associated with CTX and P1NP in female participants, whereas no significant associations were observed in male participants.^24^ Despite inconsistent links between insomnia and BMD, preliminary evidence reveals significant associations of insomnia with specific bone health markers, especially among females.

### Putative mechanisms

The hyperarousal process, a state of heightened physiologic, cortical, and cognitive-emotional activation, has attracted widespread attention as an integrative pathophysiological framework in insomnia. This state is marked by hyperactivation of the hypothalamic-pituitary-adrenal (HPA) axis with elevated cortisol levels and disrupted stress regulation.^30–32^ Excess cortisol levels, whether of endogenous or exogenous origin, disrupt bone homeostasis through direct effects on bone cells (osteoblasts, osteocytes, osteoclasts, and their precursors) and indirect effects mediated by alterations in muscle function and systemic calcium balance.^33^ In parallel, sympathetic nervous system (SNS) activation provides evidence of physiological arousal in insomnia.^34^ This insomnia-driven SNS overactivation, in concert with reduced leptin levels, profoundly alters bone metabolism via β-adrenergic receptor signaling, which promotes osteoclastogenesis through upregulation of Receptor Activator of nuclear factor-κB (NF-κB) Ligand (RANKL) while concurrently restraining bone formation through inhibition of osteoblast proliferation.^35^

Neuroendocrine factors may be implicated in the link between insomnia and osteoporosis. Insomnia-related reductions in slow-wave sleep diminish secretion of growth hormone (GH), thereby disrupting the GH/insulin-like growth factor I (IGF-I) axis.^36^ As IGF-I mediates GH’s anabolic skeletal effects by promoting osteoblast function and bone formation, this disruption impairs bone remodeling and ultimately contributes to imbalances in bone metabolism.^37^ Orexins are neuropeptides synthesized by neurons in the lateral hypothalamic area that are implicated in regulating sleep-wake homeostasis, stress resilience, reward and motivation, cognition, and feeding and metabolism.^38^ Insomnia-related hyperarousal and stress are associated with disrupted orexin secretion rhythms. The orexin system exerts dual regulation on bone homeostasis, enhancing bone formation centrally via orexin receptor 2 in the brain while inhibiting it peripherally through orexin receptor 1 in bone tissue.^39^ Thus, insomnia-induced dysregulation of the orexin system may disrupt bone homeostasis. Furthermore, insomnia interferes with the central regulation of the hypothalamic-pituitary-gonadal (HPG) axis, thus compromising the secretory rhythm and homeostasis of sex hormones.^40^ Whereas sex steroids maintain bone mass and strength by slowing the rate of bone remodeling and balancing resorption with formation, insomnia-related sex hormone disturbances disrupt this equilibrium and may further accelerate loss of bone mass.

Insomnia-induced chronic inflammation is characterized by elevated levels of tumor necrosis factor-α, interleukin-1β, and interleukin-6.^41,42^ These pro-inflammatory cytokines promote osteoclastogenesis by activating NF-κB/Nuclear Factor of Activated T-Cells Cytoplasmic (NFATc1) via RANKL-dependent and -independent pathways and suppressing osteoprotegerin (OPG). Simultaneously, they impair osteoblast differentiation by inhibiting the Wnt/β-catenin and bone morphogenetic protein (BMP) signaling pathways, thereby disrupting bone remodeling homeostasis.^43^ In parallel, the accumulation of reactive oxygen species (ROS) associated with insomnia disrupts bone homeostasis by enhancing osteoclastogenesis through impaired nuclear translocation of nuclear factor erythroid 2-related factor 2 and activation of RANKL/NF-κB signaling, while concurrently impairing osteoblast function via activation of Forkhead Box O transcription factors and suppression of the Wnt/β-catenin pathway.^44^

Insomnia also alters the diversity and composition of the gut microbiota along with the profile of microbiota-derived metabolites, and is associated with compromised intestinal mucosal barrier integrity.^45^ Accumulating evidence has highlighted the role of the microbiota-gut-bone axis in the pathophysiology of osteoporosis.^46^ Perturbations in the gut microbial community can influence bone metabolism through multiple mechanisms, including nutrient absorption, immune modulation, endocrine signaling, and gut barrier permeability. Moreover, microbial metabolites, such as short-chain fatty acids and trimethylamine N-oxide, have been implicated in the regulation of osteoblast and osteoclast differentiation.^46^ Accordingly, insomnia-induced gut dysbiosis may represent another important pathway contributing to bone metabolic disturbances.

Beyond these potential pathophysiological pathways linking insomnia to osteoporosis, their clinical association may be mediated by multidimensional frailty. A reciprocal relationship has been documented between insomnia and this frailty state, which spans physical, psychological, and social domains.^47,48^ Such integrated decline subsequently promotes osteoporotic progression via intermediary mechanisms like malnutrition, sarcopenia, and physical inactivity.^49^ In addition, the association between insomnia and osteoporosis may be further complicated by shared upstream drivers. Specifically, conditions such as cardiovascular diseases,^50,51^ metabolic syndrome,^19,52^ and depression,^53,54^ can independently contribute to both chronic insomnia and accelerated bone loss, thereby potentially explaining part of the observed epidemiological link.

### Insomnia treatments and bone health

The management of insomnia encompasses integrated approaches, positioning cognitive-behavioral therapy for insomnia (CBT-I) as the first-line intervention and pharmacotherapy as a widely implemented alternative. Although no direct evidence links CBT-I to bone metabolism, pharmacotherapy for insomnia carries potential risks to skeletal integrity. Epidemiological studies have consistently demonstrated that the use of sedative-hypnotics is associated with an elevated risk of falls and fractures. A systematic review and meta-analysis by Seppala et al. comprehensively examined the association between psychotropic medications and fall risk, revealing that benzodiazepines (BZDs) significantly increase the likelihood of both initial and recurrent falls.^55^ Moreover, several reviews have confirmed a significant association between BZD use and heightened odds of hip fractures.^56,57^ Notably, van de Ven et al. observed that the prescription of BZDs following an initial osteoporotic fracture further exacerbates the risk of subsequent fractures.^58^ Although non-BZD Z-drugs are often regarded as safer alternatives, a recent meta-analysis by Treves et al. indicated a statistically significant association between Z-drug use and an increased risk of fractures and injuries.^59^ Regarding trazodone, a common pharmacological intervention for insomnia, studies have yielded inconsistent findings on its association with fall risk, reporting both protective^60,61^ and adverse effects.^62,63^ In contrast, no significant association between orexin receptor antagonists and the risk of either falls or fractures was identified at meta-analysis by Pan et al.^64^

Beyond epidemiological associations with falls and fractures, accumulating preclinical evidence suggests that BZDs may directly influence bone metabolism through peripheral molecular targets. The translocator protein (TSPO), an evolutionarily conserved outer mitochondrial membrane protein formerly termed peripheral-type BZD receptor (PBR) due to its activation by select BZDs, has been implicated in steroidogenesis, mitochondrial homeostasis, and cellular differentiation processes, including osteogenesis.^65^ A prior study by Lee et al. confirmed the expression of TSPO in human mesenchymal stem cells (MSCs) and found that high concentrations of diazepam suppressed MSC viability.^66^ BZDs exert negative effects on the viability and osteogenic differentiation of cultured hMSCs, as evidenced by reduced alkaline phosphatase (ALP) activity and calcium deposition in vitro models.^67^ Moreover, BZDs inhibit chondrogenesis through a TSPO-dependent mechanism involving suppression of TGF-β signaling, with this effect being pharmacologically reversible through TSPO antagonist administration.^68^

For patients with insomnia, this expert consensus panel recommends the following:

- For patients with insomnia at elevated osteoporosis risk (e.g., postmenopausal women, older adults), it is recommended to assess clinical risk factors for osteoporosis or osteoporotic fracture, including menopausal status, parental hip fracture history, smoking, alcohol use, falls, and medication use. Routine BMD monitoring and the use of fracture risk assessment tools (Fracture Risk Assessment Tool (FRAX), QFracture, Garvan Fracture Risk Calculator) should be considered as appropriate.
- For patients at high or very high fracture risk (e.g., multiple vertebral fractures, severe osteoporosis, recent fragility fracture), pharmacological treatment should be initiated following specialist consultation and osteoporosis guidelines.
- Prioritize non-pharmacological interventions, with CBT-I as the first-line treatment in insomnia patients at elevated risk of osteoporosis.
- Encourage stress management, regular exercise, and healthy lifestyle practices to improve both sleep quality and bone health.
- Be cautious when prescribing BZDs and Z-drugs for insomnia patients at elevated risk of osteoporosis and ensure regular monitoring of bone health with appropriate fall-prevention measures. When pharmacological treatment is required, consider safer alternatives such as orexin receptor antagonists or melatonin receptor agonists.
- Screen for fall history. High-risk patients (recurrent falls, fall-related injuries, or inability to rise independently) should undergo a comprehensive assessment, including gait/balance testing and physical function evaluation. Consider balance, gait, and strength training for those at increased fall risk.
- Optimize the home environment to mitigate fall risks by eliminating tripping hazards and providing sufficient illumination for nighttime mobility, and to foster a sleep-conducive setting by ensuring darkness and quiet in the bedroom.
- Adopt an integrated management approach involving sleep medicine, endocrinology, and orthopedics to provide comprehensive care for patients with insomnia and comorbid osteoporosis.

## Circadian rhythm sleep-wake disorders (CRSWDs) and osteoporosis

### Epidemiology

Accumulating evidence identifies potential links between circadian rhythms and bone health. In a longitudinal study involving 402 533 UK Biobank participants followed for a median of 13.1 years, Smit et al. reported that a self-reported evening chronotype was associated with increased osteoporosis incidence and lower left femoral neck T-scores.^69^ Using data from the Korea National Health and Nutrition Examination Survey, Kim et al. reported that shift workers outside of daytime hours displayed a significantly higher risk of osteopenia compared to daytime workers. This study also revealed a significant association between night shift work and lower T scores (standard deviations from the young-adult reference mean BMD) for both the total femur and lumbar spine.^70^ A subsequent study involving 4 408 US participants suggested that night shift work was associated with an increased prevalence of clinically diagnosed osteoporosis in both the overall population and females aged ≥ 50 years, and was linked to significant decreases in total spine T score among middle-aged and older females.^71^ Similarly, a study by Quevedo et al. indicated significantly lower BMD at the lumbar spine and femoral neck among Chilean postmenopausal female nurses working rotating shifts (*n* = 39) compared to their daytime-shift counterparts (*n* = 31). The rotating-shift group also exhibited a higher proportion of osteopenia, although any statistical significance for this difference was not reported.^72^ Furthermore, Feskanich et al. examined 38 062 postmenopausal nurses in the Nurses’ Health Study and found that over 20 years of night shift work was associated with an increased risk of wrist and hip fractures during an eight-year follow-up period.^73^ In contrast, Bukowska-Damsk et al. found no significant correlation between night shift work and BMD in Polish female blue-collar workers.^74^

The literature indicates that circadian rhythms may impact bone metabolism. In a case-control study enrolling 40 nighttime shift and 42 daytime Italian healthcare personnel, Martelli et al. found significant differences in CTX and P1NP serum levels, with shift workers presenting increased levels of both bone turnover markers.^75^ In the subsequent study by Bukowska-Damsk et al., rotating night shift workers were found to have significantly higher levels of CTX, P1NP, and fibroblast growth factor 23, compared to their daytime-working counterparts.^76^ A series of experimental studies by Swanson et al. investigated the effects of combined sleep restriction with circadian disruption (SRCD) in sex- and age-stratified groups. After SRCD, female participants showed decreased levels of bone formation markers (P1NP and osteocalcin (OC)), accompanied by an increase in bone resorption marker CTX.^77^ In male participants, the intervention resulted in reduced P1NP levels, without significant changes in CTX; however, younger males exhibited elevated levels of the bone formation inhibitor sclerostin.^78,79^ Utilizing data from the Osteoporotic Fractures in Men study, Rogers et al. demonstrated that disrupted rest-activity circadian rhythms (RAR) in older men (age ≥ 65 years) were significantly associated with lower BMD at both the total hip and femoral neck, as well as increased fall risk.^80,81^ Nevertheless, no consistent or significant associations were observed between RAR patterns and fractures.^81^ Additionally, in a prospective analysis of 90 029 UK Biobank participants, Zhao et al. established RAR disturbances as an independent predictor of incident osteoporosis, with the association possibly mediated by inflammatory markers.^82^

### Putative mechanisms

The molecular circadian clock operates through transcription-translation feedback loops centered on a heterodimeric complex of brain and muscle ARNT-like 1 (BMAL1) and circadian locomotor output cycles kaput (CLOCK). This complex binds to E-box elements in target gene promoters, activating transcription of core clock components such as Period, Cryptochrome, RAR-related orphan receptors (RORs), and nuclear receptors REV-ERB. Following translation, these gene products form negative feedback loops that inhibit BMAL1/CLOCK, establishing self-sustained oscillations with near-24-h periodicity.^83^ Circadian clock genes critically regulate bone remodeling. Circadian clock genes promote osteoblast differentiation by activating core transcription factors such as Runx2 and Osterix and modulating pathways including Wnt/β-catenin and Transforming Growth Factor-β (TGF-β)/BMP.^84^ In osteoclasts, the CLOCK/BMAL1 heterodimer drives osteoclast differentiation by activating NFATc1 transcription via E-box binding, a process enhanced by steroid receptor coactivators.^85^ BMAL1 further modulates osteoclast differentiation by binding to the OPG promoter and enhancing its expression.^86^ In addition, Rev-erbα regulates osteoclastogenesis by suppressing NFATc1, and inhibits osteoclast and osteoblast differentiation through p38 mitogen-activated protein kinase (MAPK) pathway downregulation.^87^

Circadian rhythms orchestrate the temporal patterns of endocrine secretion that are essential for skeletal homeostasis. Melatonin synthesis and secretion are governed by the suprachiasmatic nucleus-driven circadian rhythm, with light serving as the primary Zeitgeber for synchronization.^88^ Functionally, melatonin exhibits multifaceted skeletal effects by concurrently promoting osteogenic differentiation while suppressing osteoclastogenesis and adipogenesis.^89^ Beyond melatonin, the parathyroid gland harbors an internal molecular circadian clock that drives diurnal variations in parathyroid hormone (PTH) secretion.^90^ PTH regulates bone remodeling by modulating calcium-phosphate and energy homeostasis, exerting anabolic or catabolic effects on bone via PTH receptor-mediated signaling cascades.^91^ Moreover, the 24-h rhythm of cortisol secretion is governed by the central circadian pacemaker, peaking at the habitual sleep-wake transition in the morning and gradually declining to its nadir in the evening.^92^ Cortisol modulates bone metabolism through integrated mechanisms, including the regulation of osteoblastogenesis, cellular apoptosis, and the skeletal microenvironment, and promotion of adipogenic differentiation in bone marrow MSCs.^93^ Furthermore, circadian misalignment can disrupt the coordinated temporal secretion of hormones and interfere with their physiological interactions. For instance, nocturnally elevated cortisol can suppress GH secretion and antagonize its bone-protective effects, ultimately compromising bone homeostasis.^94^

In addition, studies have hypothesized that circadian rhythms influence bone reconstruction by regulating energy metabolism.^95^ Circadian clocks orchestrate energy metabolism through the temporal coordination of glucose, lipid, protein and micronutrient metabolism, mitochondrial function, and hormonal rhythmicity, thereby generating metabolic oscillations that govern osteoblast differentiation, osteoclast activity, and MSC fate to maintain skeletal homeostasis. Altered feeding rhythms may further disrupt bone energy metabolism, thereby compromising skeletal homeostasis. Furthermore, circadian clocks regulate multiple facets of immune function, including immune cell trafficking, activation of innate and adaptive responses, and host-pathogen interactions.^96^ The intimate crosstalk between the immune and skeletal systems is relatively well understood, as immune cells and their secreted pro-osteoclastogenic and pro-osteogenic mediators actively participate in shaping bone remodeling.^97^ Accordingly, disruption of circadian immune regulation may therefore disturb this balance and impair bone metabolism.

### Melatonin

Preclinical evidence from in vitro and in vivo models suggests that melatonin exerts dual regulatory effects on bone remodeling by simultaneously promoting osteogenesis and inhibiting osteoclastogenesis through pleiotropic mechanistic pathways. Melatonin stimulates the proliferation, differentiation, and osteogenic gene overexpression of MSCs through the regulation of key osteogenic transcription factors and signaling pathways, including BMP/Smad, Wnt/β-catenin, and MAPK/extracellular signal-regulated kinase (ERK), as well as the modulation of non-coding RNA expression.^89^ It suppresses adipogenesis and enhances osteogenesis by downregulating peroxisome proliferator-activated receptor γ and upregulating Runx2 expression.^98^ In addition, melatonin ameliorates oxidative stress- and inflammation-induced suppression of osteogenesis, and inhibits apoptosis, thus facilitating bone formation and remodeling.^99^ Melatonin further suppresses osteoclastogenesis by inhibiting the RANKL-induced signaling cascade in a receptor-independent manner, preventing the transcriptional activation of NFATc1, a key regulator of osteoclast differentiation.^100^ Additional in vitro evidence indicates that melatonin regulates the microRNA-882/REV-ERBα axis to attenuate RANKL-induced osteoclast formation.^101^ Moreover, in a valproic acid-induced bone loss model, melatonin alleviated bone deterioration by reducing inflammation and oxidative stress, thereby improving bone metabolism.^102^

Clinical evidence indicates that oral melatonin supplementation may confer osteoprotective effects. A 6-month double-blind randomized controlled trial (RCT) by Kotlarczyk et al. involving 18 perimenopausal females observed no significant between-group differences in calcaneal BMD or bone turnover markers (N-telopeptide of type I collagen (NTX), OC); however, melatonin treatment showed a time-dependent trend toward improved bone balance, as evidenced by a decreasing NTX/OC ratio.^103^ In a subsequent RCT of 81 postmenopausal females with osteopenia in Denmark, Amstrup et al. reported that one-year of melatonin supplementation dose-dependently increased femoral neck BMD, with the higher dose also improving volumetric BMD of the spine. Melatonin further increased tibial trabecular thickness and reduced 24-h urinary calcium excretion.^104^ Maria et al. conducted an RCT in twenty-two postmenopausal women with osteopenia, establishing that MSDK (melatonin, strontium, vitamin D₃, and vitamin K₂) supplementation significantly increased lumbar spine and femoral neck BMD, with a nonsignificant upward trend at the total left hip. MSDK supplementation also elevated serum P1NP levels and decreased CTX/P1NP ratio, indicative of attenuated bone turnover.^105^ Recent findings from Piriyakhuntorn et al., including 41 middle-aged patients with iron-overloaded thalassemia, demonstrated that melatonin treatment significantly increased lumbar spine BMD, decreased serum P1NP levels, and reduced malondialdehyde concentrations, suggesting attenuation of oxidative stress.^106^ Given that endogenous melatonin levels decline with age and its deficiency is implicated in aging-related processes, melatonin supplementation for the elderly may help alleviate associated impairments and complications.^107^

For patients with CRSWDs, this expert consensus panel recommends the following:

- Perform regular assessments of skeletal health, including BMD monitoring, fracture risk, and fall history screening, particularly shift workers, night workers, and individuals experiencing chronic jet lag.
- Promote circadian realignment through structured sleep–wake scheduling (strict adherence to a pre-set timetable), a balanced and nutrient-rich diet, and outdoor physical activity.
- Apply non-pharmacological interventions such as light–dark therapy and sleep hygiene education for high-risk CRSWD patients.
- For individuals at high risk of vitamin D/calcium deficiency (older adults, limited sun exposure, falls/fracture history, chronic disease, or use of bone-active medications), consider assessing serum 25-hydroxyvitamin D and dietary calcium intake. If deficiency/insufficiency, vitamin D and calcium supplementation is recommended, with follow-up testing and regular monitoring.
- For CRSWDs, particularly specific subtypes such as delayed sleep-wake phase disorder, non-24-h sleep-wake rhythm disorder, and jet lag syndrome, melatonin use is recommended in accordance with clinical guidelines and specialist advice. Treatment should include individualized timing and dosing, safety evaluation, and regular monitoring.

## Sleep duration and osteoporosis

### Epidemiology

To promote optimal health, the American Academy of Sleep Medicine and Sleep Research Society jointly recommend that adults obtain at least 7 h of sleep per night on a regular basis.^108^ The National Sleep Foundation’s expert panel further provides age-specific recommendations: adults are recommended to sleep 7-9 h per night, whereas older adults (≥65 years) are recommended to sleep 7-8 h per night.^109^ It should be noted that individual variability in sleep needs exists, and durations marginally outside these ranges may be appropriate for some individuals.^109^ Several reviews have synthesized evidence regarding sleep durations outside the recommended range and bone health.^18,110,111^ A recent meta-analysis by Tian et al. systematically integrated 14 studies to investigate the relationship between self-reported sleep duration (defined according to the heterogeneous criteria of the included original studies), BMD, and osteoporosis across populations.^112^ In males, a U-shaped relationship was detected, where both insufficient and prolonged sleep durations were linked to an increased risk of osteoporosis. Conversely, among premenopausal and postmenopausal females, only longer sleep duration was significantly linked to osteoporosis. Notably, no significant associations were established in older adults aged 60 years and above. Extended sleep duration, but not shorter sleep duration, was significantly associated with decreased BMD in the general population.

Current research on objectively measured sleep duration and bone health remains limited. Drawing on the Study of Osteoporotic Fractures, Swanson et al. assessed sleep duration using both objective wrist actigraphy and subjective self-reports from 1 624 older postmenopausal females. Although no significant association was found between subjective sleep duration and BMD, analysis of 24-h objective sleep duration, including daytime naps, demonstrated a significant inverse association with total hip BMD.^113^ In their subsequent study, Swanson et al. reported that neither subjective nor objective sleep duration was associated with BMD or with bone turnover markers, CTX and P1NP, in older males with an average age of 77 years (*n* = 1 926), employing data from the Osteoporotic Fractures in Men study.^114^ In contrast, Petrov et al. conducted a longitudinal study of 59 English-speaking university students, which revealed that baseline average total sleep time (TST) and sleep efficiency (SE) were significantly associated with increased OC levels after a one-year follow-up. However, no significant association was observed between TST and NTX.^115^ Additionally, the study by Rassow et al. examined 1 037 German Caucasian participants and identified a significant association between polysomnography recorded TST and P1NP concentration in males after full adjustment and weighting. In contrast, TST showed no significant association with CTX levels.^24^

### Putative mechanisms

The mechanisms linking sleep duration to osteoporosis remain unclear. Nevertheless, several plausible pathways have been proposed. Physical activity is a promising agent for the prevention and management of osteoporosis.^116^ Prolonged sleep duration reduces waking time and thereby limits engagement in routine exercise. The resulting decline in physical activity leads to skeletal disuse and diminished mechanical loading, which in turn disrupts bone remodeling and promotes bone loss.^117^ Additionally, diminished outdoor activity may limit sun exposure, which can suppress endogenous vitamin D synthesis and subsequently impair bone metabolism.^118^ Moreover, excessively long or short sleep durations disrupt circadian eating rhythms, thereby promoting insulin resistance and obesity.^119^ Obesity-driven chronic inflammation and lipotoxicity exacerbate adipose tissue dysfunction, which is characterized by an imbalance in adipokine secretion (e.g., elevated leptin and decreased adiponectin), collectively leading to the impairment of bone regulation.^120^

On the other hand, established pathophysiological evidence suggests that short sleep duration may affect bone metabolism via mechanisms overlapping with insomnia and circadian disruption. Both short sleep duration and insomnia compromise sleep architecture and homeostasis, altering the proportion and continuity of sleep stages.^119^ Such disturbances initiate a shared cascade of pathophysiological processes, including activation of the HPA axis, heightened SNS activity, disruption of sex hormone secretion, metabolic dysregulation, immune suppression, and elevated inflammation^121^ and oxidative stress.^122^ In addition, sleep loss or displacement disrupts the sleep-wake cycle, which in turn feeds back onto and modulates the activity and output of the central circadian clock.^123^ This disruption propagates to peripheral tissues, leading to alterations in energy metabolism, immune and inflammatory responses, hormone signaling, and other downstream signaling pathways.^123^ Collectively, these interrelated mechanisms impair bone remodeling dynamics, ultimately promoting bone loss and increasing the risk of osteoporosis.

For patients with sleep durations outside the recommended range, this expert consensus panel recommends the following:

- Exclude secondary medical or psychiatric conditions that may affect sleep duration before initiating management.
- Distinguish between natural short/long sleepers and those with behavioral or pathological causes by assessing trajectory over time, sleep satisfaction, and daytime impairment.
- Evaluate sleep patterns comprehensively, using both objective and subjective assessments.
- Monitor bone health indicators regularly in patients with sleep durations outside the recommended range, particularly in those at elevated risk of osteoporosis. For those at elevated osteoporosis risk, consider performing fracture risk evaluation using validated tools, along with fall history screening.
- Promote the regularization of sleep-wake patterns through adequate natural light exposure, consistent meal schedules, and physical activity.
- Vitamin D and calcium status should be evaluated in high-risk individuals by measuring serum 25-hydroxyvitamin D and estimating dietary calcium intake. Supplementation is recommended when deficiency or insufficiency is identified, followed by periodic reassessment.
- Provide tailored interventions: for short sleepers with functional impairment, consider sleep hygiene education and relaxation techniques; for long sleepers with functional impairment, encourage restriction of time in bed and structured daytime physical activity.

## Obstructive sleep apnea (OSA) and osteoporosis

### Epidemiology

Mounting epidemiological findings indicate a link between OSA and compromised bone health. The latest systematic review and meta-analysis by Chan et al., which included fifteen observational studies with a total of 158 273 participants, established an inverse association between the apnea-hypopnea index (AHI) and BMD/T-scores. Specifically, individuals with OSA displayed lower BMD/T-scores and a significantly elevated risk of osteoporosis. This relationship was severity-dependent, with more severe OSA associated with greater deterioration in bone health. Notably, neither body mass index nor age were found to be significant modulator of the relationship between OSA and bone density.^17^ Emerging evidence links nocturnal hypoxemia and OSA to falls and fractures. This link may be exacerbated by OSA-associated alterations in body composition, such as sarcopenic obesity, which independently increase frailty and fall risk.^124^Analyses of the MrOS study by Cauley et al.^125^ and Stone et al.^126^ revealed that nocturnal hypoxemia (defined as peripheral oxygen saturation (SpO₂) below 90% for at least 10% of sleep time) significantly increased the risk of falls in a dose-dependent manner, whereas the AHI showed no significant association. In parallel, a study by Yilmaz Gokmen et al. of 49 Turkish OSA patients verified that both the AHI and lowest arterial oxygen saturation (SaO₂) were independent predictors of fall risk.^127^ Moreover, Kaushik et al. identified a significant association between a prior diagnosis of OSA and an increased likelihood of falls in the aging population of the Blue Mountains Eye Study.^128^ Furthermore, in a study of 28 older Australian patients with OSA, Stevens et al. demonstrated that 3-6 months of continuous positive airway pressure (CPAP) treatment significantly reduced the risk of falls.^129^ Regarding fractures, an increased risk of non-spine fracture in participants with nocturnal hypoxemia compared to those with normal nocturnal oxygen saturation was observed in a study by Cauley et al.^125^ In a subsequent analysis of the Nurses’ Health Study with a follow-up period of approximately 10 years, Huang et al. observed that female nurses with a physician-diagnosed history of OSA were at a higher risk of incident vertebral fractures than those without.^130^ In addition, Liu et al. observed a significant association between AHI and vertebral fracture prevalence in Chinese patients with a recent OSA diagnosis.^131^

Evidence also suggests that OSA is linked to altered bone turnover markers. Analyzing sixty-eight male participants (53 OSA patients vs. 15 controls), Tomiyama et al. reported significantly elevated plasma levels of IL-6 and TNF-α in the severe OSA group. They also identified a significant association between AHI and urinary CTX excretion.^132^ Following at least three months of CPAP therapy, plasma levels of interleukin-1β (IL-1β), interleukin-6 (IL-6), and tumor necrosis factor-α (TNF-α) were significantly reduced, accompanied by evidence of suppressed bone resorption, as indicated by increased plasma OPG and decreased urinary CTX.^132^ Moreover, in a study by Terzi et al. involving 30 males diagnosed with OSA and 20 healthy controls, serum β-isomerized CTX levels were significantly elevated in the OSA group, while OC levels exhibited a significant inverse correlation with AHI and mean SaO₂.^133^ In a subsequent study, Vilovic et al. demonstrated that male OSA patients (*n* = 53) had significantly lower plasma levels of bone metabolism markers, including ALP, bone-specific ALP, P1NP, OC, and β-isomerized CTX, as well as a reduced TBS compared with controls (*n* = 50). AHI was positively associated with P1NP and desphospho-uncarboxylated matrix Gla protein, and negatively correlated with TBS.^134^ Extending study to an older population, Deng et al. assessed 225 older adult OSA patients and 35 controls and found that OC levels were significantly higher in an non-OSA/mild OSA group than in the moderate-to-severe OSA group, while mean SaO₂ was negatively correlated with bone-specific ALP and positively correlated with tartrate-resistant acid phosphatase 5b.^135^ Furthermore, an investigation by Qiao et al. revealed significantly lower bone microstructural parameters, including trabecular thickness and cortical thickness, in middle-aged and older OSA patients (*n* = 80) compared with controls (*n* = 10). Additionally, a positive association was observed between AHI and trabecular thickness, whereas both the hypopnea index and the percentage TST of with SpO₂ < 90% were positively associated with ALP.^136^

### Putative mechanisms

Intermittent hypoxia (IH) from OSA induces dysregulation of hypoxia-inducible factor (HIF), which synergistically amplifies reactive ROS production and exacerbates systemic inflammation, thereby forming a vicious cycle that ultimately drives cellular injury.^137^ Under hypoxia, HIF-1α upregulation promotes osteoclast differentiation by enhancing RANKL expression via the Janus kinase 2/signal transducer and activator of transcription 3 pathway, and inhibiting ferroptosis in bone marrow-derived macrophages.^138^ In inflammatory or high-energy metabolic states, RANKL-stabilized HIF-1α promotes glycolysis and further amplifies bone resorption.^139^ Furthermore, HIF-1α regulates the fate of bone MSCs in an oxygen concentration-dependent manner, whereby mild hypoxia suppresses senescence and promotes osteogenesis, whereas severe hypoxia impairs osteogenesis and shifts differentiation toward adipogenic or chondrogenic lineages.^138^ Repetitive hypoxia-reoxygenation episodes in OSA have generally been associated with excessive generation of ROS, thereby driving systemic oxidative stress and downstream tissue damage.^140^ The physiological redox state is essential for maintaining the equilibrium between osteoblastogenesis and osteoclastogenesis, whereas oxidative stress disrupts this balance and thus impairs bone remodeling.^44^ Through synergistic interplay with HIF-1α/ROS signaling, OSA-related IH activates the proinflammatory transcription factor NF-κB, thereby initiating inflammatory cascades that culminate in systemic inflammation.^141^ As previously established, inflammation stimulates osteoclastogenesis while suppressing osteoblast differentiation, ultimately leading to bone loss.^43^ In addition to IH-driven mechanisms, sleep fragmentation (SF) associated with OSA activates stress-responsive neural circuitry,^142^ leading to dysregulation of the HPA axis, autonomic imbalance, and disruption of the hormonal milieu, including GH, sex steroids, metabolic hormones, and melatonin.^143^ Furthermore, the frequent co-occurrence of OSA with other sleep disorders, such as insomnia and CRSWDs, may further exacerbate bone remodeling imbalances via the aforementioned mechanisms. Collectively, these alterations impair bone remodeling homeostasis and thereby increase the risk of osteoporosis.

### OSA treatments and bone health

CPAP, the gold standard OSA treatment, delivers mild air pressure to ensure upper airway patency. Evidence regarding the association between CPAP therapy and bone health remains sparse. Carpi et al. demonstrated that 12 months of compliant CPAP therapy (>4 h per night) significantly increased BMD at the lumbar spine (L1-L2), in a study of 60 Italian male patients with severe OSA (AHI > 30 events/h).^144^ Tomiyama et al. reported that while patients with OSA exhibited significantly elevated urinary CTX levels, this bone resorption marker decreased after at least three months of CPAP therapy, indicating a suppression of bone resorption.^132^ In an RCT, Theorell-Haglöw et al. allocated 65 adult males with moderate to severe OSA (AHI ≥ 20 events/h) to receive either active (*n* = 34) or sham (*n* = 31) CPAP treatment. After 12 weeks of intervention, no significant between-group differences were observed in the levels of bone turnover markers, including osteocalcin, β-isomerized CTX, and P1NP. However, after 24 weeks, compliant patients (CPAP ≥ 4 h per night) showed a significant increase in osteocalcin levels.^145^ Loh et al. conducted a systematic review and meta-analysis incorporating five studies,^145–149^ which established that CPAP use did not significantly alter serum 25-hydroxyvitamin D levels.^150^ However, several of the included studies indicated potential benefits in specific subpopulations, such as males, individuals with obesity, severe OSA, or excessive daytime sleepiness.^145–147^ Moreover, some studies suggested that long-term or highly compliant CPAP use may lead to improvements in vitamin D status, particularly among patients with low baseline levels.^145,148^ Notably, two subsequent studies have reported that long-term CPAP use can increase vitamin D levels in patients with severe OSA and in those with OSA comorbid with chronic obstructive pulmonary disease.^144,151^ Additionally, the antioxidant and anti-inflammatory effects of CPAP may also contribute positively to bone metabolism.^152^ Therefore, we hypothesize that long-term or high compliance to CPAP therapy could be beneficial for skeletal health. For CPAP-nonadherent patients, second-line OSA management includes surgery, mandibular advancement devices, and pharmacotherapy with agents such as carbonic anhydrase inhibitors and incretin-based therapies.^153,154^ However, current evidence on their skeletal effects remains limited. Studies have reported that certain carbonic anhydrase inhibitors may possess bone-sparing effects, alongside a dose-dependent risk of metabolic acidosis disrupting calcium-phosphate homeostasis.^155^

For patients with OSA, this expert consensus panel recommends the following:

- Monitor bone health and assess fall/fracture risk regularly in patients with confirmed or suspected OSA, particularly those with moderate-to-severe disease.
- Adopt individualized weight management. Monitor and mitigate bone health sequelae of rapid weight loss and underweight.
- Incorporate resistance training and balanced nutrition to preserve bone mass. For individuals.
- with insufficient serum 25-hydroxyvitamin D levels or inadequate dietary calcium intake, vitamin D and calcium supplementation is recommended, with follow-up testing and regular monitoring.
- Recommend CPAP therapy as the standard treatment for OSA, and promote compliance through regular follow-up, patient education, and supportive care to optimize both respiratory and skeletal outcomes.
- For CPAP-nonadherent patients, consider tailored therapies such as surgery, mandibular advancement devices, or pharmacotherapy, while minimizing sedentariness and monitoring drug dosage and adverse effects.
- Adopt a multidisciplinary approach involving respiratory and sleep medicine, endocrinology, and orthopedics to provide individualized and comprehensive care for OSA patients at elevated risk of osteoporosis.

## Other categories of sleep disorders and osteoporosis

Evidence linking other sleep disorders to osteoporosis remains sparse. Restless legs syndrome (RLS) is a sensorimotor disorder characterized by a rest-induced, evening-worsening urge to move the legs that is relieved by movement.^156^ In a cross-sectional case-control study of 78 treatment-naïve women with RLS and 78 age- and BMI- matched controls, Cikrikcioglu et al. found that the RLS group had significantly higher lumbar BMD and lower levels of CTX and sclerostin.^157^ The authors hypothesized that the involuntary, symptom-driven movements of RLS might serve as a form of passive mechanical loading, thereby mitigating bone loss.^157^ Narcolepsy is a rare neurological disorder characterized by selective loss or dysfunction of hypocretin-producing neurons in the lateral hypothalamus.^158^ Reis et al. observed a significant positive correlation between cerebrospinal fluid hypocretin-1 levels and bone mineral content percentage in a comparative study of 31 type 1 and 18 type 2 narcolepsy patients.^159^ In a longitudinal cohort study, Chang et al. reported that narcolepsy patients (*n* = 493) had a significantly elevated risk of bone fractures compared to matched controls (*n* = 490) after average follow-up periods of 8.0 and 9.7 years, respectively.^160^

## Conclusions

Epidemiological, mechanistic, and interventional evidence systematically demonstrates that insomnia, CRSWDs, sleep durations outside the recommended range, and OSA are each significantly associated with an increased risk of osteoporosis. Based on these associations, this consensus report provides clinical recommendations for bone health in patients with sleep disorders and emphasizes the integration of sleep disorder management into osteoporosis prevention and care pathways, offering new perspectives for both primary and secondary prevention. Further preclinical and longitudinal clinical studies and RCTs are needed to validate these associations, clarify underlying mechanisms and pathways, and establish multidisciplinary collaborative strategies.

## Supplementary information

Supplementary Tables

Supplementary Figure

## Registration information

This CONSENSUS STATEMENT has been registered with the Practice guideline REgistration for transPAREncy (PREPARE), with the registration number PREPARE-2026CN553.
