# Supplementary material for: Expert consensus on osteoporosis risk management in patients with sleep disorders
Source: Bone Res. 2026 Aug 13;14:85. doi: 10.1038/s41413-026-00562-0 (PMC13473140; doi:10.1038/s41413-026-00562-0)
Supplement: Supplementary file 1 [file 41413_2026_562_MOESM1_ESM.docx]

**Supplementary Table 1.** Study characteristics of observational studies on sleep disorders and osteoporosis.

| Author, year | Country/ Region | Study design | Follow-up (y) | Sample size, % Female | Average age (y) | Sleep disorder assessment | Osteoporosis assessment | BMD assessment site | BTMs assessment | Adjusted/Matched covariates |
| --- | --- | --- | --- | --- | --- | --- | --- | --- | --- | --- |
| insomnia and osteoporosis | | | | | | | | | | |
| Sivertsen et al., 2014 | Norway | Prospective cohort | 11 | 24715, 56.9% | 45.3 | Clinically diagnosed | Self-reported | NA | NA | Age, sex, education, anxiety, depression, angina, arthrosis, asthma, ankylosing spondylitis, cancer, diabetes, fibromyalgia, headache, hypertension, myocardial infarction, obesity (BMI > 30), osteoporosis, rheumatoid arthritis, stroke, whiplash |
| Yen et al., 2014 | Taiwan | Retrospective cohort | 12 | 134070, 60.7% | 51.5 | Clinically diagnosed | Clinically diagnosed | NA | NA | Age, sex, diabetes, hypertension, hyperlipidemia, CKD, stroke, COPD, cancer, anxiety, depression benzodiazepine use, zolpidem use |
| Cherian et al., 2022 | India | Prospective cohort | 2.5 | 190, 100.0% | 58.2 | WHIIRS | ISCD guidelines | BMD at lumbar spine (L1-L4) and femur neck | CTX, P1NP | Previous history of fracture, duration since menopause |
| Pengpid et al., 2024 | Thailand | Prospective cohort | 5 | 2863, 55.6% | NR | Self-reported | Clinically diagnosed | NA | NA | Age, sex, education, marital status, subjective economic status, residential area, religion |
| Rassow et al., 2024 | Germany | Cross-sectional | NA | 1037, 45.2% | 52.6 | ISI/PSQI | Self-reported | NA | CTX, P1NP | Age, sex, BMI, alcohol consumption, smoking, physical inactivity |
| Zhao et al., 2024 | China | Prospective cohort | 13.7 | 293164, 52.0% | 56.4 | Self-reported | Clinically diagnosed | NA | NA | Sex, age, smoking, alcohol, tea, coffee consumption, BMI, IPAQ, Townsend deprivation index, risk of OSA, depression |
| Zhou et al., 2023 | China | Cross-sectional | NA | 289000, 54.0% | 56.7 | Self-reported | NA | BMD at heel and whole body | NA | Age, sex, BMI, assessment centers, alcohol intake and smoking status, serum vitamin D levels, physical activity |
| Niu et al., 2015 | China | Cross-sectional | NA | 750, 72.4% | 58.8 | Self-reported | NA | BMD at femoral neck, trochanter, total hip, and lumbar spine (L2-L4) | NA | Age, educational level, smoking status, drinking status, BMI, physical activity score, hypertension, diabetes, arthritis, plasma 25(OH)D concentration, snoring frequency, osteoporosis medication use, CES-D, menopause status |
| Yamaura et al., 2023 | Japan | Cross-sectional | NA | 1717, 47.8% | 54.5 | Self-reported | NA | BMD at right calcaneus | NA | Age, menopausal status, early menopause, BMI, physical activity, smoking, alcohol intake, diabetes mellitus, dyslipidemia |
| Pan et al., 2020 | Australia | Prospective cohort | 10.7 | 1099, 52.0% | 62.9 | Self-reported (Sleep disruption) | NA | BMD at hip, lumbar spine and whole body | NA | Age, sex,BMI, physical activity, smoking history, the presence of any comorbidities, multisite pain |
| CRSWDs and osteoporosis | | | | | | | | | | |
| Smit et al., 2025 | The Netherlands | Prospective cohort | 13.1 | 402533, 55.2% | 56.6 | Self-reported chronotype | Clinically diagnosed | BMD at left femoral neck, lumbar spine and total radius | NA | Sex, age, BMI, Townsend deprivation index, smoking status, alcohol use, corticosteroid use, sleep medication use, bisphosphonate use, use of vitamin D and/or calcium, use of HRT, oral contraceptive use, handedness |
| Kim et al., 2013 | South Korea | Cross-sectional | NA | 3005, 48.0% | 36.4 | Shift work | Clinically diagnosed | BMD at lumbar spine, total femur, and femoral neck | NA | Age, sex, BMI, education, marital status, residential area, household income, current smoking, current alcohol, regular physical activity, daily intake of calcium and phosphate, serum 25(OH)D |
| Qian et al., 2024 | China | Cross-sectional | NA | 4408, 47.2% | 43.5 | Shift work | Clinically diagnosed | BMD at femoral neck and total spine | NA | Age, sex, ethnicity, BMI, blood pressure, HbA1c, diabetes, eGFR, CKD |
| Quevedo et al.,  2010 | Chile | Cross-sectional (Comparative) | NA | Shift: 39 vs. Daytime: 31, 100.0% | Shift: 55.6 vs. Daytime: 55.9 | Shift work | NA | BMD at lumbar spine (L1-L4) and both hips | NA | NA |
| Bukowska-Damsk et al., 2020 | Poland | Cross-sectional | NA | 194, 100.0% | 49.5 | Shift work | Clinically diagnosed | BMD at lumbar spine, total femur, and femoral neck | NA | BMI, ever-used HRT, smoking |
| Martelli et al., 2023 | Italy | Cross-sectional | NA | Shift: 42 vs. Daytime: 40, 0.0% | Shift: 30.5 vs. Daytime: 34.0 | Shift work | NA | NA | CTX, P1NP | Age, smoking |
| Bukowska-Damsk et al., 2022 | Poland | Cross-sectional | NA | 189, 100.0% | 49.5 | Shift work | NA | NA | CTX, P1NP, FGF-23, OC, OPN, OPG | BMI, menopausal status |
| Rogers et al., 2017 | USA | Prospective cohort | 4 | 2412, 0.0% | 75.7 | RAR | NA | BMD at total hip and femoral neck | NA | Amplitude, pseudo-F-statistic, mesor: BMI, heart attack, diabetes, alcohol, caffeine, PASE, depression, melatonin, beta blocker use, 25(OH) D, urinary calcium. Alpha and beta: BMI, smoking, alcohol, depression. Acrophase: race, BMI, melatonin, alcohol, smoking, caffeine, PASE, depression. |
| Zhao et al., 2025 | China | Prospective cohort | 8.1 | 90029, 55.5% | 62.2 | RAR | Clinically diagnosed | NA | NA | Sex, age, ethnicity, recruitment regions, educational attainment, smoking, alcohol, body mass index, Townsend deprivation index, physical activity, and history of diabetes, long-standing illness, hypertension, cancer |
| Sleep duration and osteoporosis | | | | | | | | | | |
| Swanson et al., 2019 | USA | Cross-sectional | NA | 874, 100.0% | 83.3 | Objective sleep duration (wrist actigraphy) | NA | BMD at total hip and femoral neck | NA | Age, race, clinical site, BMI, Calcium/Vitamin D use, depression, walking speed, HTN, COPD, daily naps, hours of sleep needed to feel rested, estrogen use |
| Swanson et al., 2020 | USA | Cross-sectional | NA | 1055, 0.0% | 76.6 | Objective sleep duration (wrist actigraphy) | NA | BMD at total hip, femoral neck, and lumbar spine | CTX, P1NP | Age, race, clinical site, BMI, alcohol, 25(OH)D level, eGFR, daily naps, hours of sleep needed to feel rested, melatonin level, calcium use, androgen use, anti-androgen use, PASE Score, self-reported health status |
| Petrov et al., 2024 | USA | Cross-sectional Exploratory longitudinal | 1 | 59, 51.8% | 20.4 | Objective sleep duration (wrist actigraphy) | NA | BMD at total femur and lumbar spine (L1-L4) | OC, NTX-1 | Age, sex, race, ethnicity, BMI, dietary intake, calcium intake, daily total activity count, season of assessment |
| OSA and osteoporosis | | | | | | | | | | |
| Tomiyama et al., 2008 | Japan | Cross-sectional (Case-control) Interventional | NA | Case: 50 vs. Control: 15, 0.0% | Case: 53.0 vs. Control: 51.4 | PSG | NA | NA | CTX, sRANKL, OC, OPG, BALP | Age, BMI, SBP, DBP, TC, TG, HDL-C, FPG, smoking status and medication status for hypertension, diabetes mellitus and/or dyslipidemia |
| Terzi et al., 2015 | Turkey | Cross-sectional (Case-control) | NA | Case: 30 vs. Control: 20, 0.0% | Case: 52.4 vs. Control: 50.6 | PSG | NA | BMD at lumbar spine and femoral neck | CTX, OC, BALP | NA |
| Vilovic et al., 2020 | Croatia | Cross-sectional (Matched case-control) | NA | Case: 53 vs. Control: 50, 0.0% | Case: 50.4 vs. Control: 51.8 | PSG | NA | BMD at lumbar spine (L1-L4), non-dominant femoral neck and total hip | CTX, P1NP, OC, BALP | Age, sex, BMI |
| Deng et al., 2024 | China | Cross-sectional | NA | 260, 44.5% | 71.3 | HSAT | Clinically diagnosed | BMD at lumbar vertebrae (L1-L4) and bilateral hip | CTX, P1NP, OC, BALP, TRAP5b | Age, sex, BMI, hypertension, smoking |
| Qiao et al., 2024 | China | Cross-sectional (Case-control) | NA | Case: 80 vs. Control: 10, 0.0% | Case: 47.3 vs. Control: 45.6 | PSG | Clinically diagnosed | HR-pQCT BMD at tibia and radius (non-dominant side) | CTX, ALP | NA |
| Other sleep disorders and osteoporosis | | | | | | | | | | |
| Cikrikcioglu et al., 2016 | Turkey | Cross-sectional (Matched case-control) | NA | Case: 78 vs. Control: 78, 100.0% | Case: 46.5 vs. Control: 45.0 | Clinically diagnosed RLS | NA | BMD at lumbar spine (L1-L4) and left proximal femur | CTX, P1NP, sclerostin | Matched: Age and BMI; Adjusted: Age at menarche, number of births, number of miscarriages or curettage, duration of breastfeeding, employment status, smoking, calcemia, phosphatemia, creatinine, albumin, 25(OH)D, transferrin saturation rate, duration of disease, and IRLSSG severity score, presence of menopause, BMI, monthly income of family |

25(OH)D: 25-hydroxyvitamin D; ALP: alkaline phosphatase; BALP: bone-specific alkaline phosphatase; BMD: bone mineral density; BMI: body mass index; BTMs: bone turnover markers; CES-D: Center for Epidemiologic Studies Depression Scale; CKD: chronic kidney disease; COPD: chronic obstructive pulmonary disease; CRSWDs: circadian rhythm sleep-wake disorders; CTX: C-terminal telopeptide of type I collagen; DBP: diastolic blood pressure; eGFR: estimated glomerular filtration rate; FGF-23: fibroblast growth factor-23; FPG: fasting plasma glucose; HbA1c: glycated hemoglobin; HDL-C: high-density lipoprotein cholesterol; HR-pQCT: high-resolution peripheral quantitative computed tomography; HRT: hormone replacement therapy; HSAT: home sleep apnea testing; HTN: hypertension; IPAQ: International Physical Activity Questionnaire; IRLSSG: International Restless Legs Syndrome Study Group; ISI: Insomnia Severity Index; ISCD: International Society for Clinical Densitometry; NA: not applicable/not available; NR: not reported; NTX-1: N-terminal telopeptide of type I collagen; OC: osteocalcin; OGTT: oral glucose tolerance test; OPG: osteoprotegerin; OPN: osteopontin; OSA: obstructive sleep apnea; P1NP: procollagen type I N-terminal propeptide; PASE: Physical Activity Scale for the Elderly; PSG: polysomnography; PSQI: Pittsburgh Sleep Quality Index; RAR: rest-activity rhythm; RLS: restless legs syndrome; SBP: systolic blood pressure; sRANKL: soluble receptor activator of nuclear factor kappa-B ligand; TC: total cholesterol; TG: triglycerides; TRAP5b: tartrate-resistant acid phosphatase 5b; WHIIRS: Women’s Health Initiative Insomnia Rating Scale.

**Supplementary Table 2.** Grading of Recommendations, Assessment, Development, and Evaluation (GRADE) for observational studies on sleep disorders and osteoporosis.

| Association | Studies, n | Study design | Certainty assessment | | | | | Certainty |
| --- | --- | --- | --- | --- | --- | --- | --- | --- |
|  |  |  | Risk of bias | Inconsistency | Indirectness | Imprecision | Publication bias |  |
| Insomnia and Osteoporosis risk | 6 | Observational | Not serious | Serious | Not serious | Not serious | Not serious | Very low |
| Insomnia and BMD | 5 | Observational | Not serious | Not serious | Not serious | Serious | Not serious | Very low |
| Insomnia and BTMs | 2 | Observational | Not serious | Not serious | Not serious | Serious | Not serious | Very low |
| CRSWDs and Osteoporosis risk | 4 | Observational | Not serious | Not serious | Not serious | Serious | Not serious | Very low |
| CRSWDs and BMD | 5 | Observational | Not serious | Serious | Not serious | Not serious | Not serious | Very low |
| CRSWDs and BTMs | 2 | Observational | Serious | Not serious | Not serious | Serious | Not serious | Very low |
| RAR and osteoporosis risk | 1 | Observational | Not serious | NA | Not serious | Not serious | Undetected | Very low |
| RAR and BMD | 1 | Observational | Not serious | NA | Serious | Not serious | Undetected | Very low |
| Objective sleep duration and BMD | 3 | Observational | Not serious | Not serious | Serious | Serious | Not serious | Very low |
| Objective sleep duration and BTMs | 3 | Observational | Not serious | Not serious | Serious | Serious | Not serious | Very low |
| OSA and BTMs | 5 | Observational | Serious | Not serious | Not serious | Serious | Not serious | Very low |
| RLS and BMD | 1 | Observational | Not serious | NA | Serious | Not serious | Undetected | Very low |
| RLS and BTMs | 1 | Observational | Not serious | NA | Serious | Not serious | Undetected | Very low |

BMD: bone mineral density; BTMs: bone turnover markers; CRSWDs: circadian rhythm sleep-wake disorders; RAR: rest-activity rhythm; OSA: obstructive sleep apnea; RLS: restless legs syndrome.
