# Supplementary material for: Expert consensus on osteoporosis risk management in patients with sleep disorders
Source: Bone Res. 2026 Aug 13;14:85. doi: 10.1038/s41413-026-00562-0 (PMC13473140; doi:10.1038/s41413-026-00562-0)
Supplement: Supplementary file 2 [file 41413_2026_562_MOESM2_ESM.docx]

**Supplementary Figure 1.** Putative mechanisms linking sleep disorders and osteoporosis. (A) Mechanisms between insomnia and osteoporosis. (B) Mechanisms between CRSWDs and osteoporosis. (C) Mechanisms between sleep durations outside the recommended range and osteoporosis. (D) Mechanisms between OSA and osteoporosis. HPA: hypothalamic-pituitary-adrenal axis; SNS: sympathetic nervous system; HPG: hypothalamic-pituitary-gonadal axis; GH: growth hormone; CRSWDs: circadian rhythm sleep-wake disorders; TTFLs: transcription-translation feedback loops; OSA: obstructive sleep apnea; HIF-1α: hypoxia-inducible factor-1α
